# Interactions of the amphiphiles arbutin and tryptophan with phosphatidylcholine and phosphatidylethanolamine bilayers in the dry state

**DOI:** 10.1186/2046-1682-6-9

**Published:** 2013-07-24

**Authors:** Antoaneta V Popova, Dirk K Hincha

**Affiliations:** 1 Max-Planck-Institut für Molekulare Pflanzenphysiologie, Am Mühlenberg 1, D-14476 Potsdam, Germany; 2 Institute of Biophysics and Biomedical Engineering, Bulgarian Academy of Sciences, 1113 Sofia, Bulgaria

**Keywords:** Amphiphiles, Arbutin, Desiccation, Fourier-transform infrared spectroscopy, Lipid phase transition, Model membranes, Tryptophan

## Abstract

**Background:**

Water is essential for life, but some organisms can survive complete desiccation, while many more survive partial dehydration during drying or freezing. The function of some protective molecules, such as sugars, has been extensively studied, but much less is known about the effects of amphiphiles such as flavonoids and other aromatic compounds. Amphiphiles may be largely soluble under fully hydrated conditions, but will partition into membranes upon removal of water. Little is known about the effects of amphiphiles on membrane stability and how amphiphile structure and function are related. Here, we have used two of the most intensively studied amphiphiles, tryptophan (Trp) and arbutin (Arb), along with their isolated hydrophilic moieties glycine (Gly) and glucose (Glc) to better understand structure-function relationships in amphiphile-membrane interactions in the dry state.

**Results:**

Fourier-transform infrared (FTIR) spectroscopy was used to measure gel-to-liquid crystalline phase transition temperatures (T_m_) of liposomes formed from phosphatidylcholine and phosphatidylethanolamine in the presence of the different additives. In anhydrous samples, both Glc and Arb strongly depressed T_m_, independent of lipid composition, while Gly had no measurable effect. Trp, on the other hand, either depressed or increased T_m_, depending on lipid composition. We found no evidence for strong interactions of any of the compounds with the lipid carbonyl or choline groups, while all additives except Gly seemed to interact with the phosphate groups. In the case of Arb and Glc, this also had a strong effect on the sugar OH vibrations in the FTIR spectra. In addition, vibrations from the hydrophobic indole and phenol moieties of Trp and Arb, respectively, provided evidence for interactions with the lipid bilayers.

**Conclusions:**

The two amphiphiles Arb and Trp interact differently with dry bilayers. The interactions of Arb are dominated by contributions of the Glc moiety, while the indole governs the effects of Trp. In addition, only Trp-membrane interactions showed a strong influence of lipid composition. Further investigations, using the large structural diversity of plant amphiphiles will help to understand how their structure determines the interaction with membranes and how that influences their biological functions, for example under freezing or dehydration conditions.

## Background

While water is generally a pre-requisite of life, some species of microorganisms, plants and invertebrates are able to survive complete desiccation (anhydrobiosis; [1]). Many more species are able to survive partial dehydration, e.g. due to extracellular ice crystallization during freezing. Common and well documented responses to cellular dehydration are increases in the amounts of compatible solutes such as sugars and some amino acids, and the accumulation of stress-protective proteins such as late embryogenesis abundant (LEA) and heat shock proteins (see e.g. [2-4] for reviews).

While the function of these compounds has been widely investigated, the potential function of small amphiphilic molecules in anhydrobiosis and dehydration stress tolerance has received much less attention. Many of these compounds, such as flavonoids, are closely related to plant freezing and drought stress tolerance [5,6], but their function has been mainly discussed in terms of their strong anti-oxidant properties [7]. Amphiphiles are generally found mainly in the aqueous phase of cells under fully hydrated conditions, but reversibly insert into membranes with decreasing water availability [8]. The degree of insertion dependends not only on water availability, but also on the polarity of the substances and potentially on the lipid composition of the target membranes. The interactions may influence membrane stability, but it is so far not possible to predict the effect of a given amphiphile on the stability of a particular membrane [9,10].

In the present study we used two particularly well-investigated amphiphiles, tryptophan (Trp) and arbutin (Arb), to elucidate the details of their interactions with membranes upon desiccation and the effects of these interactions on the physical state of the membranes. Arb (4-hydroxyphenyl-β-D-glucopyranoside) has been found in high concentrations in the leaves of the desiccation tolerant resurrection plant *Myrothamnus flabellifolia* from South Africa [11,12]. Arb is an antioxidant [13] and inhibits phospholipase A_2_ in partially dehydrated membranes [14]. It interacts directly with membranes already in the fully hydrated state [15], but membrane stabilization during freezing or drying requires the presence of non-bilayer lipids such as phosphatidylethanolamine (PE) or monogalactosyldiacylglycerol (MGDG) [16,17]. In the presence of these lipids, Arb relaxes the inverted curvature stress and thereby stabilizes the bilayer state during dehydration [10,18].

The interactions of the aromatic amino acid Trp with membranes have attracted wide-spread attention, because it is thought to anchor membrane-spanning proteins at the membrane-water interface [19,20]. While free Trp shows no measurable interactions with membranes in the fully hydrated state [21], it destabilizes both chloroplast membranes and liposomes during freezing, independent of the lipid composition [22].

Since both the hydrophobic (indole and phenol) and hydrophilic (amino acid and glucose) moieties of Trp and Arb are quite different, distinct interactions with and effects on membranes could be expected. To distinguish the contributions of the hydrophobic and hydrophilic parts of the two molecules, we investigated in addition the effects of glycine (Gly) and glucose (Glc). Fourier-transform infrared (FTIR) spectroscopy was used to monitor the effects of the additives on membranes containing two typical phospholipids, namely the bilayer lipid phosphatidylcholine (PC) and the non-bilayer lipid PE. The analysis of specific vibration bands of the additives provided additional information on their interactions with the lipids and on the role of particular functional groups in the different molecules. A previous detailed investigation of the lipid systems employed in this study has already shown that within the temperature limits employed here (up to 70°C), all membranes are exclusively in a liquid-crystalline bilayer configuration and that no non-bilayer transitions were detectable [23].

## Methods

### Liposome preparation

Egg phosphatidylcholine (EPC), egg phosphatidylethanolamine (EPE) and 1,2 dimyristoyl-sn-glycero-3-phosphocholine (DMPC) were obtained from Avanti Polar Lipids (Alabaster, AL). Trp and Arb were obtained from Fluka, Gly and Glc from Sigma.

Liposomes were made from either pure EPC, pure EPE, or 50% EPE/50% EPC or 50% EPE/50% DMPC on a weight basis. Lipids (10 mg) were dried from the solvent under a gentle steam of N_2_. Residual chlorophorm was removed under vacuum overnight. The resulting lipid film was hydrated in 200 μl H_2_O or in 50 mM solutions of Arb, Trp, Glc or Gly. Large unilamellar liposomes were formed with a hand-held extruder (Avestin, Ottawa, Canada; [24]) with two layers of 100 nm pore size polycarbonate membranes. Samples (50 μl) were spread on CaF_2_ windows and dried in desiccators over silica gel at 28°C for six to eight hours and subsequently kept under vacuum overnight. Since pure EPE does not form bilayers, 2.5 mg lipid in chloroform were spread directly on CaF_2_ windows, the solvent was evaporated under a stream of N_2_ and then under vacuum over night [23].

### Fourier-transform infrared (FTIR) spectroscopy

FTIR measurements were performed using a Perkin-Elmer GX 2000 Fourier-transform infrared spectrometer as described previously [25,26]. A CaF_2_ window with a dry sample was fixed in a cuvette holder placed in a vacuum chamber with a temperature control unit (Specac Eurotherm, Worthington, UK) and placed in the infrared beam. Sample temperature was monitored by a fine thermocouple, attached to the surface of the window, next to the sample. Samples were kept under vacuum for 30 min at 30°C to remove residual water absorbed during handling. The absence of an absorbance band at 1650 cm^-1^ and the position of the asymmetric P = O stretching vibration at 1262 cm^-1^ (100% EPC) indicated that samples were essentially anhydrous [23,27]. Temperature was decreased to –30°C and after equilibration for 10 min the temperature was increased to 70°C with a constant rate of 1°C/min. Infrared absorbance spectra were recorded between 4000 and 900 cm^-1^ and analysed using the Spectrum 2000 software (PerkinElmer). The band positions of characteristic molecular group vibrations were analyzed after normalization of absorbance and baseline correction of the spectra by the interactive ABEX and FLAT routines of the software.

Positions of characteristic peaks originating from lipid molecules (compare [27-29]), CH_2_ symmetric stretching (νCH_2_s), C = O stretching (νC = O), asymmetric P = O stretching (νP = Oas) and asymmetric choline N^+^(CH_3_)_3_ stretching (νN^+^(CH_3_)_3_as) vibrations were determined by the automatic peak identification routine. Hydrogen bonding interactions of the OH groups of Glc and Arb were analyzed through the broad peak of the OH stretching band (νOH) in the spectral region of 3600 – 3000 cm^-1^[30]. The vibration peaks arising from the indole part of Trp (3430 – 3380 cm^-1^), from the amino part of Trp and Gly (1690 – 1550 cm^-1^) and from the phenol group of Arb (1530 – 1490 cm^-1^) were also used to determine interactions between lipids and additives. Except for the νCH_2_s vibration, all peaks were analysed at 70°C where all lipid membranes were in the liquid-crystalline phase (compare Table 1). The gel to liquid-crystalline phase transition temperatures (T_m_) of the lipids in the absence and presence of additives were determined as the midpoints of the lipid melting curves monitored as the temperature dependent increase in the position of the νCH_2_s peak [29]. Interactions at the level of C = O esters were estimated by analyzing the νC = O stretching peak by deconvolution using Origin7.0 [27,31].

**Table 1:** **Gel to liquid-crystalline phase transition temperatures (T**_**m **_**in °C) of dry liposomes in the absence (pure) or presence of Arb, Glc, Trp, or Gly**

**Lipid composition**	**Pure**	**Arb**	**Glc**	**Trp**	**Gly**
**100% EPC**	40	−24	−24	20	40
**100% EPE**	−3	−18	−18	5	−2
**50% EPE/**	0	−22	−15	20	2
**50% EPC**					
**50% EPE/**	48	−20	−13	29	51
**50% DMPC**					

**Figure 1:**
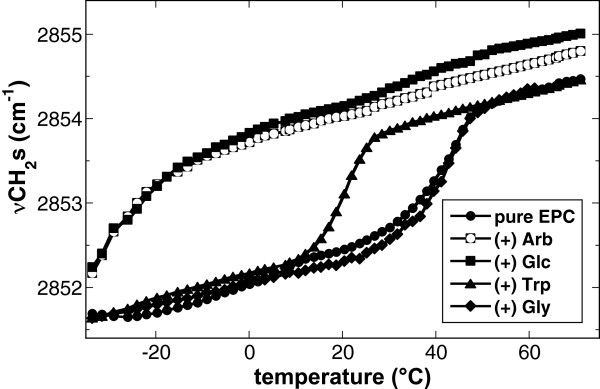
**Temperature induced increase of νCH**_**2**_**s of dry liposomes made from EPC in the absence or presence of the additives Arb, Glc, Trp or Gly.** Liposomes were formed either in water or, to have the additives present on both sides of the membranes, in 50 mM solutions of the respective additives. Phase transition temperatures (T_m_) from gel to liquid-crystalline state were determined as the midpoint of the melting curves. T_m_ values for all samples are shown in Table 1.

T_m_ was determined by FTIR spectroscopy as the midpoint of melting curves obtained as shown in Figure 1. Data for the pure lipids are identical to those published previously [23].

## Results

### Membrane phase behavior

The physical state of lipid bilayers can be monitored by the temperature-induced shift of the νCH_2_s peak to higher wavenumbers, as this vibration is sensitive to changes in the conformational disorder of the hydrocarbon chains [29]. The midpoint of this transition is defined as the gel to liquid-crystalline phase transition temperature (T_m_). Figure 1 shows the effects of the additives Arb, Glc, Trp and Gly on the melting curves of EPC liposomes after drying. Dry EPC showed a T_m_ of 40°C (Table 1), while the addition of either Arb or Glc depressed T_m_ by more than 60°C to -24°C. In the presence of Trp the T_m_ of EPC was decreased more moderately by 20°C, while Gly had no measurable influence. Phase transition temperatures for all other lipids (pure EPE, 50% EPE/50% EPC, 50% EPE/50% DMPC) in the absence and presence of all additives are presented in Table 1. Arb and Glc caused a large depression of T_m_ for all investigated lipids. This ranged from 15–20°C for EPE and EPE/EPC membranes to 60–70°C for EPE/DMPC. The effects of Trp on T_m_ were generally smaller (10–20°C), with increases for EPE and EPE/EPC and decreases for EPC and EPE/DMPC. Gly, on the other hand, did not influence the T_m_ of any of these lipids.

The destabilizing effect of Trp on model membranes and in particular the induction of vesicle fusion during freezing, has been suggested earlier to be partially due to the formation of hexagonal (H_II_) phase [22]. DSC measurements, however, provided no evidence for Trp-induced formation of H_II_ in any of the investigated membrane systems in the dry state (data not shown).

### Interactions in the interfacial headgroup region

Since the effects of the additives on lipid phase transitions varied widely both between additives and lipids, it was of interest to investigate possible solute-lipid interactions in more detail.

**Figure 2:**
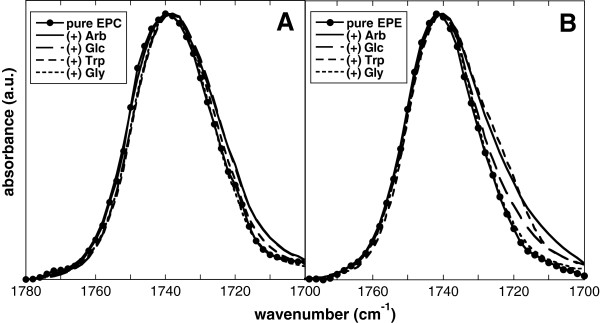
**Normalized infrared peaks of the νC = O vibration from dry bilayers.** EPC **(A)** and EPE **(B)** liposomes were dried in the absence or presence of Arb, Glc, Trp or Gly and FTIR spectra were obtained at 70°C.

The carbonyl ester groups (C = O) of diacyl lipids are situated between the hydrophobic fatty acyl chains and the hydrophilic headgroup region. In the dry state, they are potential H-bonding partners for the headgroups of neighbouring lipid molecules and for any additives. The C = O groups give rise to an infrared vibration peak centered at around 1738 cm^-1^. As described in detail in a previous publication [23], the C = O peak position varied by several wavenumbers with lipid composition (Figure 2). However, the different additives had no influence on the peak position. Nevertheless, in EPE both Arb and Trp induced a peak broadening towards the low-wavenumber side, indicating a low degree of interaction with the C = O groups. This was also observed for EPE/EPC and EPE/DMPC liposomes (data not shown), but not for pure EPC (Figure 2). Glc only had a very small effect, while spectra recorded in the presence of Gly were in all cases identical to those obtained with the pure lipids.

The C = O peak is composed of two component bands arising from free and H-bonded C = O groups. The higher wavenumber component at about 1742 cm^-1^ is attributed to non-H-bonded (free) C = O groups, while the lower wavenumber component at about 1726 cm^-1^ originates from H-bonded C = O groups [32-34]. The ratio between the areas under the two component peaks is proportional to the relative fraction of C = O groups involved in H-bonding [27,31]. However, deconvolution of the C = O peaks revealed the expected differences between the different lipid compositions [23], but only minor effects of the additives (data not shown), again indication only a low degree of interactions.

**Figure 3:**
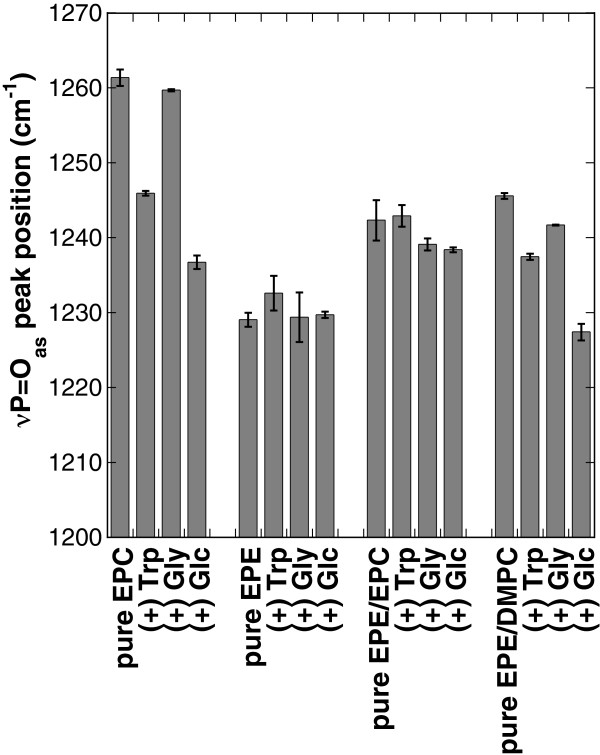
**Position of the νP = Oas vibration peak from dry bilayers.** Liposomes were made from pure EPC, pure EPE, 50% EPE/50% EPC, or 50% EPE/50% DMPC in the presence or absence of Trp, Gly or Glc. FTIR spectra were obtained at 70°C. Data represent the means ± SE from at least three samples.

The hydrophilic headgroups of the phospholipids in this study contain the phosphate (P = O), and choline (N^+^(CH_3_)_3_) or ethanolamine (N^+^CH_3_) moieties. The P = O group is characterized by an infrared asymmetric vibration peak between 1300 and 1200 cm^-1^. This peak is located at about 1260 cm^-1^ for anhydrous PC and can shift to around 1220 cm^-1^ depending on the extent of H-bonding to e.g. water or sugars [28,34,35]. Figure 3 shows the positions of νP = Oas from the different lipids in the absence and presence of Trp, Gly or Glc. Unfortunately, the effect of Arb on νP = Oas could not be investigated due to overlap with a prominent peak originating from the phenol part of Arb (1260 – 1170 cm^-1^).

For all lipids in the absence of additives the νP = Oas peak was situated between 1261 cm^-1^ for EPC and 1230 cm^-1^ for EPE, in agreement with previous publications [23,36]. In the presence of Trp the P = O peak of pure EPC was shifted from 1261 cm^-1^ to 1246 cm^-1^, indicating that Trp was H-bonded to the P = O groups of the lipid. Addition of Gly to EPC liposomes, on the other hand, had no clear influence on νP = Oas (peak shift of less than 2 cm^-1^). Such a slight shift or no shift in the peak position was also observed for the other lipids, indicating only a very low level of H-bonding between P = O groups and Gly even in the dry state. The largest shift by 24 cm^-1^ was observed in νP = Oas of EPC in the presence of Glc. This position was 8 cm^-1^ lower than in the presence of Trp, indicating that the P = O groups were involved in more H-bonding interactions, in agreement with the general propensity of sugars to H-bond to anhydrous lipids [35,37].

For EPE and EPE/EPC all investigated additives had no or only a very sligh effect on νP = Oas. The maximum effect for EPE/EPC was about 4.5 cm^-1^ and for EPE about 3 cm^-1^, indicating that the P = O groups preferentially interacted with ethanolamine groups rather than with the additives [23,36]. Stronger effects of Trp and Glc on νP = Oas were found for EPE/DMPC. Here, νP = Oas was shifted by 8 cm^-1^ with Trp and by 18 cm^-1^ with Glc.

The terminal part of the PC headgroup is the choline moiety that gives rise to an asymmetric stretching vibration at around 970 cm^-1^, that is sensitive to H-bonding interactions with water [26,38] or sugars [27,39]. When the choline group is involved in such interactions, its vibration is shifted by a maximum of 4 cm^-1^ to higher wavenumbers [27,39]. Here, we generally observed shifts in νN^+^(CH_3_)_3_as of less than 1 cm^-1^ (data not shown), indicating that the additives only weakly interact with the choline group.

### Interactions between lipids and additives as revealed by IR peaks derived from the additives

An important advantage of infrared spectroscopy is that every chemical group in a sample gives rise to specific vibrations, providing simultaneous information on different components of a mixture. The substances investigated in the present study are all characterized by typical peaks in the FTIR spectra that allowed the evaluation of interactions between additives and membrane lipids.

**Figure 4:**
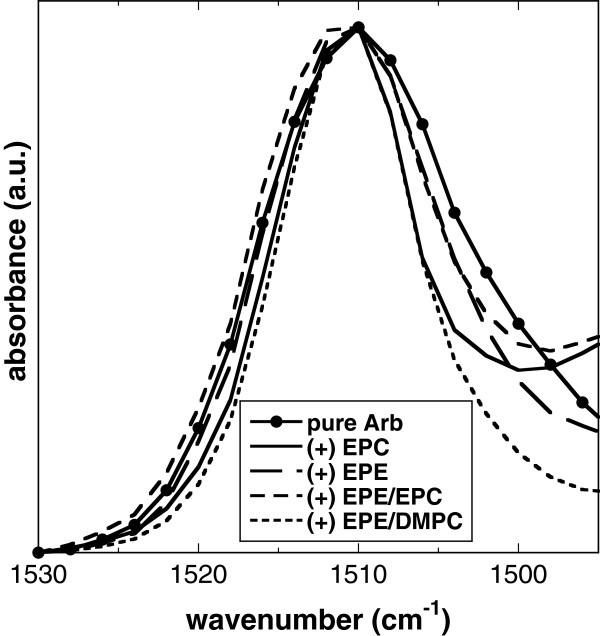
**Normalized infrared vibration peak from the phenol moiety of dry Arb.** Arb was dried in the absence or presence of liposomes made from pure EPC, pure EPE, 50% EPE/50% EPC, or 50% EPE/50% DMPC. Spectra were recorded at 70°C.

The phenol moiety of Arb is characterized by two peaks in the IR regions of 1260–1170 cm^-1^ and 1530–1490 cm^-1^. As mentioned above, the lower wavenumber peak overlaps with νP = Oas of phospholipids and was therefore not suitable for the evaluation of phospholipid-Arb interactions. The second peak, originating from the vibration of aromatic double bonds, was centered at about 1510 cm^-1^ for dry Arb in the absence and presence of liposomes (Figure 4), in agreement with published data [40]. The presence of liposomes, however, resulted in peak narrowing. Peak width at half-height was reduced from 17 cm^-1^ for pure Arb to 12.5 cm^-1^ in the presence of EPC, 14 cm^-1^ with EPE and EPE/EPC, and 10.5 cm^-1^ with EPE/DMPC, indicating a more restricted range of vibrations in the presence of lipid, in agreement with the proposed localization of the phenol moiety of Arb in the membranes [15].

**Figure 5:**
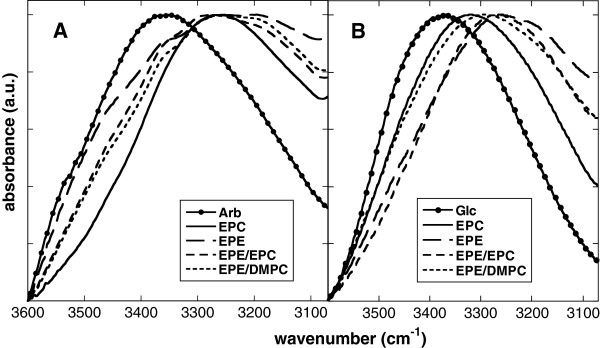
**Normalized νOH peaks originating from OH groups of the Glc moiety of dry Arb (A) or of free Glc (B).** The solutes were dried in the absence or presence of liposomes made from pure EPC, pure EPE, 50% EPE/50% EPC, or 50% EPE/50% DMPC. Spectra were recorded at 70°C.

Interactions between Glc, either in the free form or as a part of the Arb molecule, and membrane lipids was monitored through the OH stretching vibration (νOH) between 3600 and 3000 cm^-1^. The position of νOH provides information about the length and strength of H-bonds formed by OH groups of Glc [30], with peaks at lower wavenumbers indicating shorter and stronger H-bonds. The νOH peak of dry Glc was situated at 3368 cm^-1^ (Figure 5), in good agreement with a published value of 3360 cm^-1^[41]. The νOH peak of dry Arb was located at 3372 cm^-1^, indicating a similar physical environment for Glc in both cases. The presence of liposomes shifted the peak downfield by around 100 cm^-1^ indicating a strong H-bonding network between Glc and the membrane lipids both for free Glc and for the Glc in Arb. The similar positions of νOH in both cases indicate H-bonding networks of similar strengths.

**Figure 6:**
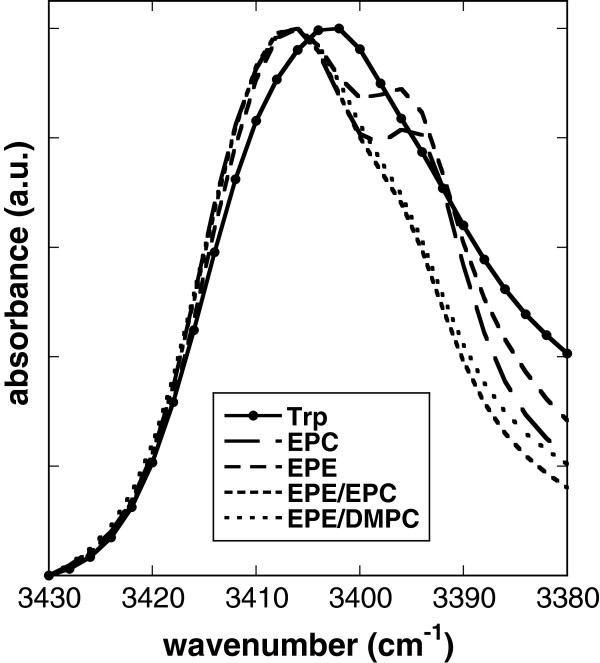
**Normalized infrared vibration peaks of the indole moiety of Trp.** Spectra were recorded at 70°C from Trp dried in the absence or presence of liposomes made from pure EPC, pure EPE, 50% EPE/50% EPC, or 50% EPE/50% DMPC.

The aromatic amino acid Trp shows two characteristic absorbance peaks in the FTIR spectrum. A peak between 3430 and 3380 cm^-1^ is attributed to vibrations from the indole part of the molecule [42,43] and a double peak, originating from the amino part, in the region between 1690 and 1550 cm^-1^ is also found in the Gly spectrum. The indole peak of pure dry Trp was located at 3402 cm^-1^ (Figure 6). In the presence of liposomes this peak was shifted to 3406 cm^-1^, irrespective of the lipid composition. In the presence of pure EPC or EPE liposomes there was a clear shoulder at about 3396 cm^-1^, which was hardly visible in the mixed membranes.

**Figure 7:**
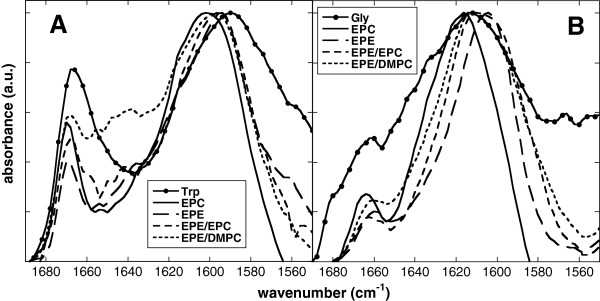
**Normalized infrared vibration peaks of the amino groups of dry Trp (A) or Gly (B).** The amino acids were dried in the absence or presence of liposomes made from pure EPC, pure EPE, 50% EPE/50% EPC, or 50% EPE/50% DMPC. Spectra were recorded at 70°C.

The two peaks originating from the amino groups of the amino acids were located at 1668 and 1590 cm^-1^ in the case of Trp and at 1662 and 1614 cm^-1^ for Gly (Figure 7). In the presence of liposomes both Trp maxima were shifted, the high-wavenumber peak by 2 cm^-1^ and the low-wavenumber peak between 4 cm^-1^ for pure EPE and 12 cm^-1^ for pure EPC. The high-wavenumber peak of Gly was relatively smaller than the corresponding Trp peak and was not consistently influenced by the presence of lipids. The low-wavenumber peak of Gly, on the other hand, showed a significant narrowing in the presence of all lipids and a shift to lower wavenumbers in the presence of EPE (pure EPE by 10 cm^-1^, EPE/EPC by 4 cm^-1^, EPE/DMPC by 2 cm^-1^).

## Discussion

### The effect of Arb on dry bilayers is dominated by Glc-lipid interactions

Sugars can preserve the structural and functional integrity of membranes in the anhydrous state [35,37]. This has been extensively investigated with disaccharides such as sucrose and trehalose. However, also monosaccharides such as Glc are able to depress the T_m_ of PC in the dry state [44]. The T_m_ of dry EPC is 40°C [26,44] and it was depressed to -24°C by both Arb and Glc, while for EPE it was shifted from about 0°C [44] to -18°C by both additives. A depression of T_m_ in dry PC by Arb has been reported previously [15]. Interestingly, the T_m_ of both EPE/EPC and EPE/DMPC was about 7°C lower in the presence of Arb than Glc, indicating that Glc fixed to the membrane surface by the phenol moiety of Arb may under some conditions be more effective than free Glc. A similar conclusion was reached for free and lipid-bound di-galactose in a previous study [45].

The decrease of T_m_ of dry lipids in the presence of sugars is related to the ability of sugars to H-bond with their OH groups to the P = O headgroups of phospholipids, as demonstrated by a shift of νP = Oas to lower wavenumbers (see e.g. [35,37] for comprehensive reviews). Here, we showed a down-shift of νP = Oas of dry EPC by 24 cm^-1^ in the presence of Glc. Unfortunately, νP = Oas does not provide information about H-bonding interactions between Arb and lipid P = O groups due to the overlap with the peak originating from the phenol moiety of Arb. However, indirect information about such interactions can be gained from the analysis of the *v*OH vibrations of Arb and Glc. The νOH peaks from both substances showed the same massive down-shift by about 100 cm^-1^ in the presence of liposomes, indicating that Arb depressed T_m_ by the same mechanism as free Glc, i.e. by H-bonding of Glc OH groups to the P = O groups of the lipids.

For EPE liposomes, the position of the P = O peak is only slightly affected by the presence of Glc, which could indicate the absence of H-bonding interactions. However, we note that the position of νP = Oas for anhydrous EPE (1230-1234 cm^-1^) [23,46] is also only shifted by about 15 cm^-1^ from the fully hydrated state [47,48], while the shift is about 40 cm^-1^ for PC [34]. This indicates that even in the presence of excess water P = O groups of PE preferentially interact with the lipid ethanolamine groups. These strong interactions persist also in mixed PE/PC liposomes [23]. In all cases, the ethanolamine headgroups of PE are involved in a tight H-bonding network with the P = O groups of phospholipids [49].

The present data suggest that when Glc is added to fully hydrated liposomes, OH groups of Glc compete with ethanolamine groups for H-bonding to P = O groups upon the removal of water. We cannot evaluate to what extent P = O groups are involved in H-bonds with either ethanolamine or Glc, but it is reasonable to assume that P = O groups will preferentially be H-bonded with OH groups of Glc rather than with ethanolamine groups as O-H · · · O bonds are stronger than C-H · · · O bonds [30].

In addition to the Glc, also the phenol part of Arb interacts with lipid bilayers, as shown by fluorescence spectroscopy with hydrated membranes [15] and by the narrowing of the corresponding FTIR peak in the dry state reported here. While the interaction with Arb-bound Glc has the same effect on T_m_ of both bilayer and non-bilayer forming lipids, the effects on liposome stability measured as carboxy fluorescein (CF) leakage or membrane fusion, are different. While liposomes made from PC are strongly destabilized during freezing or drying, the inclusion of non-bilayer lipids such as PE or MGDG results in membrane stabilization by Arb [15-17]. It has been argued that Arb destabilizes membranes containing only bilayer lipids due to the partitioning of its phenol moiety into the membrane-water interface, thus eliciting positive membrane curvature stress. On the other hand, when membranes contain non-bilayer lipids, these cone-shaped molecules [50] induce negative curvature stress that will be further increased on removing water [51]. In this case, the insertion of Arb into the membrane interface reduces the negative curvature stress and stabilizes the lipids in a bilayer configuration [16,17].

### The effect of Trp on dry bilayers is dominated by indole-lipid interactions

Trp decreased T_m_ for EPC and EPE/DMPC liposomes, while for EPE and EPE/EPC the transition from gel to liquid-crystalline phase occurred at temperatures higher than for the pure lipid bilayers. Interestingly, the influence of Trp on liposome stability, measured as either CF leakage or membrane fusion, was independent of lipid composition. Trp destabilized membranes made from pure EPC and from EPE/EPC, although the effect was more pronounced for membranes containing EPE [22]. Gly, on the other hand, had no measurable influence on T_m_, regardless of lipid composition, in agreement with our earlier data for EPC [42]. This indicates that unlike the Glc in Arb, the hydrophilic amino acid part of Trp plays no important role in Trp-membrane interactions in the dry state. This is in agreement with the fact that the vibration peak attributed to the amino part of Trp and Gly was not changed in its position in the presence of membranes. However, in the presence of liposomes this peak was more cooperative than for the pure substances, indicating restricted vibration. In addition, the presence of Gly induced only a small shift in νP = Oas, in agreement with only minor interactions.

The shift in νP = Oas induced by Trp in EPC was considerably larger, but we suggest that it was not due to interactions with the amino acid moiety. It has been shown both experimentally and in Molecular Dynamics (MD) simulations that indole or Trp in small peptides localize mainly in the interfacial region between the first two to three carbons of the hydrocarbon chains and the choline group in fully hydrated PC membranes [52-58]. In agreement with our data, earlier studies indicate the possibility of interactions between the indole NH group and lipid P = O, while interactions with the lipid C = O groups are of minor importance [52,55,56,58].

Analogous to what we have discussed above for Glc and Arb, we suggest that also the interactions between Trp and EPE involve H-bonding to P = O groups, although the position of the νP = Oas peak is not strongly influenced. Again, this may be explained by competition of the indole NH groups with ethanolamine groups for bonding to P = O groups. This is supported by a shift of the peak attributed to the Trp indole ring by about 4 cm^-1^ in the presence of liposomes, independent of lipid composition, indicating similar interactions with EPC and EPE. This is in agreement with MD simulations that found similar interactions of Trp in gramicidin A with either PE or PC bilayers [57].

One of the most striking effects that we observed in this study is the opposite direction of changes in T_m_ in the presence of Trp, depending on lipid composition. While T_m_ was decreased for dry EPC and EPE/DMPC by 20°C, it was increased by 8°C and 20°C for EPE and EPE/EPC, respectively. The decrease of T_m_ in EPC membranes by Trp can be easily explained on the basis of published data. Both experimental and MD studies have shown that the indole ring is fairly rigid and oriented parallel to the fatty acyl chains in lipid bilayers [52,53,55,58]. The localization of the indole ring near the glycerol backbone leads to interactions with the upper part of the fatty acyl chains, reducing molecular mobility in this part of the lipid molecules. At the same time, this results in a wider spacing of the lipids and increased space and mobility in the lower parts of the chains that can be observed as a decrease in T_m_ in our FTIR experiments. Unfortunately, there are no comparative data or simulations for PE lipids in the literature. However, it can be reasonably speculated that the cone shaped PE molecules will allow a deeper penetration of the indole ring into the fatty acyl chain region of the bilayers due to the formation of geometrical defects, as recently characterized in an MD simulation study [59]. This could limit chain mobility sufficiently to increase T_m_, while the effect of EPE could be counterbalanced by DMPC. The fully saturated fatty acyl chains may lead to stronger interactions of the rigid indole ring with this lipid [52] than with EPE, again inducing higher mobility in the lower chain region. However, this hypothesis needs to be tested in future experiments and MD simulations.

## Conclusions

In conclusion, we have shown in this study that the two amphiphiles Arb and Trp have different modes of interaction with dry bilayers. While the interactions of Arb are completely dominated by the contribution of the hydrophilic Glc moiety, the hydrophobic indole determines the effects of Trp. In addition, there was only a small effect of lipid composition on Arb-bilayer interactions, while a pronounced influence of lipid composition was obvious for Trp-membrane interactions. Further investigations, that could e.g. use the large structural diversity of flavonoids, which are also known to efficiently interact with lipid bilayers [60-63], will be needed to understand how amphiphile structure determines the interaction with membranes. In addition, such investigations could shed light on the functional relevance of the large natural diversity in the structure of these molecules and relate it to biological functions, such as plant freezing and dehydration tolerance.

## Competing interests

The authors declare that they have no competing interests.

## Authors’ contributions

AVP designed and conducted all experiments, analyzed the data and drafted the manuscript. DKH participated in experimental design and data analysis, and wrote the final manuscript. Both authors read and approved the final manuscript.
